# Supplementary material for: Tensor-network approach for quantum metrology in many-body quantum systems
Source: Nat Commun. 2020 Jan 14;11:250. doi: 10.1038/s41467-019-13735-9 (PMC6959326; doi:10.1038/s41467-019-13735-9)
Supplement: Supplementary file 1 — Supplementary Information [file 41467_2019_13735_MOESM1_ESM.pdf]

Supplementary Information for: Quantum Metrology in Many-Body Quantum Systems—a Tensor Network Approach

Chabuda et al.

SUPPLEMENTARY NOTE 1. CHARACTERIZATION OF METROLOGICAL MODELS WHERE EFFICIENT TREATMENT WITH MPO APPROACH IS POSSIBLE

Consider a system comprised of N distinguishable d-dimensional particles, so that the total Hilbert space is $\mathcal{H} = \bigotimes_{n=1}^{N} \mathbb{C}^d$. We assume that the parameter φ is unitarily encoded in the output state ρ_φ according to a product of unitaries given by the exponential of local generators (or Hamiltonians):

$$\rho_\varphi = \Lambda_\varphi(\rho_0) = \mathrm{e}^{-\mathrm{i}H\varphi}\Lambda(\rho_0)\mathrm{e}^{\mathrm{i}H\varphi}, \quad H = \sum_{n=1}^{N} h^{[n]}, \tag{1}$$

where $h^{[n]}$ is the generator acting on the nth particle.

We assume that the noise operator Λ may be effectively approximated as a product of single ($\Lambda^{[n]}$), two- ($\Lambda^{[n,n+1]}$), three- ($\Lambda^{[n,n+1,n+2]}$), etc. particle maps up to some cut-off point after which to is assumed that noise correlations do not extend beyond r neighbouring particles. From a more physical perspective, consider a time-independent quantum master equation[1] describing the noisy part of the evolution of an N-body quantum system:

$$\frac{\mathrm{d}\rho}{\mathrm{d}t} = \sum_{k=1}^{r}\sum_{n=1}^{N} \mathcal{L}^{(k,n)}(\rho), \tag{2}$$

$$\mathcal{L}^{(k,n)}(\rho) = \sum_j \mathcal{D}\left[L_j^{[n,\dots,n+k-1]}\right](\rho), \tag{3}$$

where

$$\mathcal{D}[c](\rho) = c\rho c^\dagger - \frac{1}{2}\left(\rho c^\dagger c + c^\dagger c\rho\right). \tag{4}$$

In the above, the $L_j^{[n,\dots,n+k-1]}$ are noise operators acting on k neighbouring particles (not to be confused with the SLD), and the combined effect of the k-particle noise acting on the subset $[n, n+1, n+k-1]$ of particles is represented by the $\mathcal{L}^{(k,n)}$ operator, where we neglect terms with higher range than r—note that a three-particle term may in particular represent a two-body interaction between next-nearest neighbours. The channel Λ can now be obtained by integrating the evolution over some fixed time t:

$$\Lambda = \exp\left(\sum_{k=1}^{r}\sum_{n=1}^{N}\mathcal{L}^{(k,n)}t\right). \tag{5}$$

If all the operators in the above exponent commute we can immediately write Λ as a product of single, two-, three- etc. maps acting on different subsets of particles, which will lead us immediately to an efficient MPO description of the dynamics, see Results section. Otherwise, one may approximate the evolution for a time t as a product of short time steps, where in each time step we perform the Suzuki-Trotter decomposition[2–4].

In what follows we often use a vectorized density matrix notation $\rho \to |\rho\rangle$, which is useful in the MPO approach, and where clear from context, we switch between the cases where Λ is understood as acting on ρ or $|\rho\rangle$. For example, $|\Lambda(\rho_0)\rangle = \Lambda|\rho_0\rangle$.

For definiteness, we assumed above that the noise acts before the unitary encoding. This entails no loss of generality if the noise commutes with the encoding, as is the case in the most popular metrological models of phase/frequency estimation in presence of dephasing or loss[5–7]. If needed, one can extend our formalism to the case where the parameter dependence no longer commutes. This comes at the expense of a slightly higher complexity of formulas and numerics, since we are no longer able to write the derivative of ρ_φ over the parameter as a commutator with the Hamiltonian. Instead, the entire channel structure Λ_φ determines the derivative.

Note that, thanks to the assumptions of the model, if the input probe state ρ_0 is only locally correlated, which is a sufficient condition for an efficient MPO description, then it remains so under the above evolution. Moreover, the derivative of the output state with respect to the parameter φ—required for calculations of the QFI—reads $\rho'_\varphi = \mathrm{i}\,[\rho_\varphi, H]$ and, since H is the sum of local Hamiltonians, is also be efficiently describable using MPO.

Most importantly, in essentially all realistic metrological protocols, the maximal achievable QFI scales linearly with N in the limit of large particle numbers[6,7], and the quantum-enhancement advantage appears in the form of a constant factor. This further implies that it is enough to consider input states ρ_0 with finite-range correlations to achieve almost optimal metrological performance[8]. As a result the MPO formalism is ideally suited to tackle this class of metrological problems and guarantees that the optimal values of QFI as well as the optimal probe states will be found via this approach.

SUPPLEMENTARY NOTE 2. EXTENDED DISCUSSION OF MAGNETIC FIELD SENSING WITH FLUCTUATING NOISE

For a better physical insight into the model, let us provide the corresponding master equation in the form of Eq. (2). One may obtain it by simply differentiating Eq. (10) over t. As a result we get the master equa-

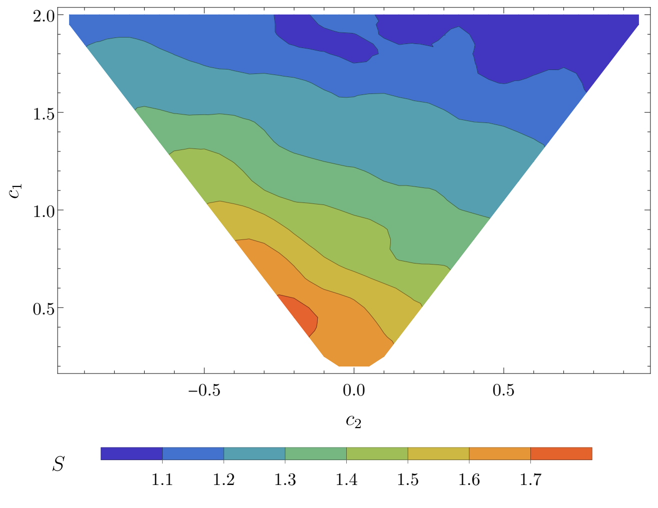

Supplementary Figure 1. Von Neumann entropy of the optimal state. Entanglement of the optimal iMPO state quantified via the Von Neumann entropy (in bits) of the reduced density matrix (obtained by tracing out half of the spin chain) of the optimal state (obtained using iMPO approach) as a function of c_1 and c_2 noise parameters.

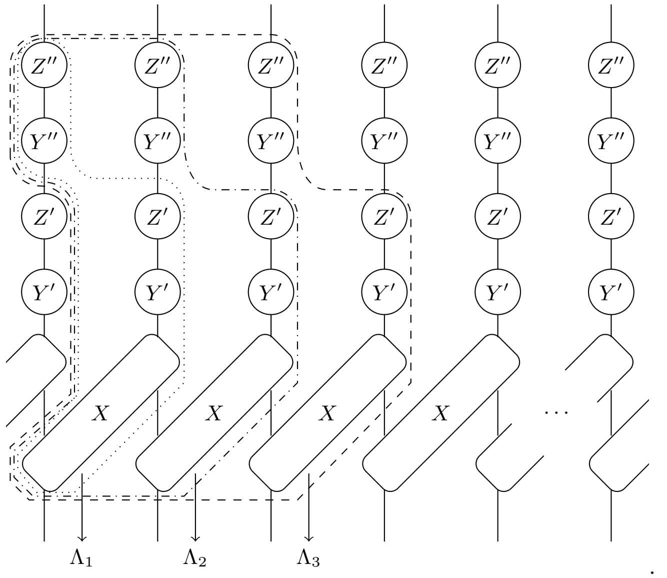

Supplementary Figure 2. Quantum channel decompositions. Different ways of rewriting the locally correlated noise dynamics as a collection of independent quantum channels: Λ_1 (dotted), Λ_2 (dash-dotted), Λ_3 (dashed).

tion with single particle $L^{[n]} = \sqrt{\gamma_1} h^{[n]}$ and two particle $L^{[n,n+1]} = \sqrt{|\gamma_2|}(h^{[n]} + \mathrm{sgn}(\gamma_2) h^{[n+1]})$ dephasing operators. The dephasing rates γ_1, γ_2 are related with field fluctuation properties as follows: $\gamma_1 = (\sigma^2 - 2|\chi|)g^2$, $\gamma_2 = \chi g^2$—note that $\sigma^2 \geq 2|\chi|$ by virtue of positivity of the correlation matrix C so the rate γ_1 is always positive.

As a supplement to the numerical results presented in Fig. 1(b) of the main text, and in order to appreciate the amount of entanglement that is present in the optimal states, in Supplementary Figure 1 we provide a contour plot of the results of calculation of the von Neuman entropy for the iMPO[9] corresponding to the reduced density matrix of the system when half of the particles is traced out (optimal QFI can be achieved by many different states so some fluctuations of entropy are to be expected). We see a clear relation, between the amount of entanglement and the increase in sensing precision.

Let us now ask the question, whether some insight into the problem could have been gained by ingeniously adapting the state-of-the-art methods of deriving fundamental bounds in quantum metrology developed with uncorrelated noise models in mind[6,7,10–12]. These methods provide easily calculable asymptotic bounds, based on just the knowledge of the Kraus operators of elementary probe dynamics or alternatively noise jump operators appearing in the quantum master equation. Even though the noise in our problem is correlated we can formally divide the evolution as a collection of independent channels acting on two, three or more particles—a similar trick has been employed in a recent study of the impact of many-body effects in atomic interferometry[13]. In the scheme below we indicate the possible constructions. First we formally decompose local dephasing gates Y as products $Y = Y'Y''$ of local dephasing gates with corresponding c_1' and c_1'' such that $c_1 = c_1' + c_1''$, and the unitary encoding gate Z as a product $Z = Z'Z''$, where Z', Z'' are phase gates with corresponding phases φ', φ'', such that $\varphi = \varphi' + \varphi''$. We can now unravel the total dynamics as effectively composed of N independent channels Λ_1, or group the gates into $N/2$ larger channels Λ_2, or $N/3$ channel Λ_3, etc.—see Supplementary Figure 2. We may now apply the bounds derived for uncorrelated noise models[7,10] using either decomposition of the dynamics into Λ_1, Λ_2 or Λ_3 channels—we can group the operations in any way we please as in this case all elementary evolutions commute. In order to obtain the tightest bound we numerically optimize the split of phases as well as noise contributions between gates Y', Z' and Y'', Z'', while making sure that the resulting Λ_i is a legitimate quantum channel—note that the bare two qubit gate X is not a proper quantum channel as it is not completely positive. The results obtained are depicted in the left inset of Fig. 1(b). While the bounds are tight for the decorrelated noise model they are far from the actual achievable QFI in the correlated (or anti-correlated) noise regimes and as expected they improve when we increase the elementary channel size. Still, because this method scales badly with the elementary channel size we were forced to stop with Λ_3 channel. This demonstrates, that state-of-the-art methods developed with uncorrelated noise models in mind yield bounds that are far from satisfactory in case of correlated noise models.

We now discuss in more detail the issue of asymptotic saturability of the bounds using weakly spin squeezed

states. For concreteness, consider the following one-axis squeezed state of N particles:

$$|\psi\rangle = \mathrm{e}^{i\theta S_z^2}|+\tfrac{1}{2}\rangle^{\otimes N}, \tag{6}$$

where θ is the squeezing strength. We follow the standard protocol[14], where the above state is rotated to the equator of the Bloch sphere so that the $\langle\vec{S}\rangle$ points in the x direction, in a way that the direction in which the angular momentum has minimal variance is y. The state is then subject to locally correlated dephasing evolution and is rotated by an unknown angle φ. Assuming we operate around $\varphi \approx 0$, we measure the S_y observable as this is the optimal choice in this case, from which value we infer the value of φ. Using the standard linear error propagation formula the resulting uncertainty of estimating the phase reads: $\Delta\widetilde{\varphi} = \sqrt{\Delta^2 S_y}/\left|\frac{\mathrm{d}\langle S_y\rangle}{\mathrm{d}\varphi}\right|$. In order to calculate the above quantity we move to the Heisenberg picture. Since we operate around $\varphi = 0$, we can replace $\frac{\mathrm{d}\langle S_y\rangle}{\mathrm{d}\varphi} = \langle S_x\rangle$, and plug $\varphi = 0$ everywhere. Under the locally correlated dephasing noise the relevant expectation values should be replaced according to the following rules:

$$\begin{aligned}
&\langle s_x^{[n]}\rangle \to \langle s_x^{[n]}\rangle \mathrm{e}^{-\frac{1}{2}c_1}, \langle s_y^{[n]}\rangle \to \langle s_y^{[n]}\rangle \mathrm{e}^{-\frac{1}{2}c_1} \\
&\langle s_y^{[n]} s_y^{[n+2]}\rangle \to \langle s_y^{[n]} s_y^{[n+2]}\rangle \mathrm{e}^{-c_1} \\
&\langle s_y^{[n]} s_y^{[n+1]}\rangle \to \langle s_y^{[n]} s_y^{[n+1]} \cosh c_2 + s_x^{[n]} s_x^{[n+1]} \sinh c_2\rangle \mathrm{e}^{-c_1}.
\end{aligned} \tag{7}$$

Taking the limit $N \to \infty$, $\theta \to 0$ in a way that $N\theta^2 \ll 1$ we obtain that the relevant expectation values on the squeezed state read[14] (assume $\hbar = 1$): $\langle s_x^{[n]}\rangle \to \frac{1}{2}\mathrm{e}^{-c_1}$, $\langle s_y^{[n]}\rangle = 0$, $\langle s_y^{[n]} s_y^{[m]}\rangle \to -\frac{1}{4(N-1)}$, $\langle s_x^{[n]} s_x^{[m]}\rangle \to \frac{1}{4}$ $(n \neq m)$. As a result $\langle S_x\rangle = \frac{N}{2}\mathrm{e}^{-c_1}$ while

$$\begin{aligned}
\Delta^2 S_y = \langle S_y^2\rangle = \frac{1}{4}N - \frac{\mathrm{e}^{-c_1}}{4(N-1)}(N-2)(N-1) \\
+ 2(N-1)\mathrm{e}^{-c_1}\left(\frac{1}{4}\sinh c_2 - \frac{1}{4(N-1)}\cosh c_2\right).
\end{aligned} \tag{8}$$

This leads to the final formula for the asymptotic precision $\Delta\widetilde{\varphi} = \sqrt{(1 - \mathrm{e}^{-c_1} + 2\mathrm{e}^{-c_1}\sinh c_2)/(N\mathrm{e}^{-c_1})}$ which can be related with the corresponding Fisher information per particle equal to:

$$\frac{F}{N} = \frac{\mathrm{e}^{-c_1}}{1 - \mathrm{e}^{-c_1} + 2\mathrm{e}^{-c_1}\sinh c_2} \tag{9}$$

as quoted in the main text.

Had we considered a product state strategy, the only modification in the above reasoning would be a substitution $\langle s_y^{[n]} s_y^{[m]}\rangle = 0$ $(n \neq m)$, which would lead to the corresponding QFI per particle:

$$\frac{F_{\mathrm{prod}}}{N} = \frac{\mathrm{e}^{-c_1}}{1 + 2\mathrm{e}^{-c_1}\sinh c_2}, \tag{10}$$

which also agrees perfectly with the numerical results as can be seen in Supplementary Figure 3.

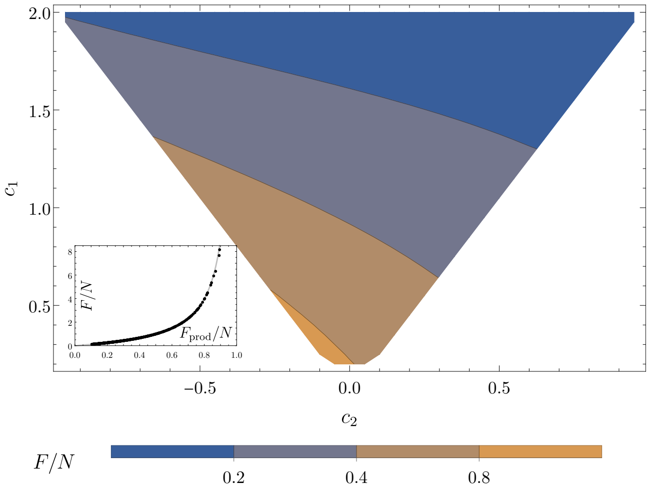

Supplementary Figure 3. Quantum Fisher Information for a product input state. Asymptotic value (obtained using the iMPOs) of QFI per particle for product input states ($D_\psi = 1$) as a function of c_1, c_2 noise parameters. Inset shows the dependence of the optimal QFI per particle as a function of the corresponding product state QFI per particle F_{prod}/N, revealing the functional dependence known from the uncorrelated dephasing case $\frac{F}{N} = \frac{F_{\mathrm{prod}}}{N}/\left(1 - \frac{F_{\mathrm{prod}}}{N}\right)$.

SUPPLEMENTARY NOTE 3. EXTENDED DISCUSSION OF ATOMIC CLOCK STABILIZATION BOUNDS

Typically we will think of the d dimensional spaces of atomic states as mentioned in the main text as being a fully symmetric subspace of $d-1$ two-level systems representing the relevant clock transition levels of the atoms—therefore we will use the notation for the states $|\psi\rangle = \sum_{k=0}^{d-1} a_k|k\rangle$, which correspond to the symmetric state, where k atoms are in an excited state and $d-1-k$ atoms in the ground state. Interaction between the LO and the atomic sample has form of Ramsey interferometry and after a single interrogation step will effectively encode the phase in the above written state as $|\psi\rangle_T = \sum_{k=0}^{d-1} a_k \mathrm{e}^{-ik\int_0^T \delta\omega(t)\mathrm{d}t}|k\rangle$, where $\delta\omega(t)$ is the detuning of the LO frequency from the atomic reference frequency ω_0. The effect of LO fluctuations is equivalent to collective dephasing of atoms and hence the state will remain within the symmetric subspace—note that we are talking about the single Hilbert space $\mathcal{H}$ here which will be represented by single node in the MPO framework. The key feature from our perspective is the fact that the LO frequency fluctuations are temporally correlated, and hence the collective dephasing acting on atoms at different interrogation steps (in our representation different steps are formally represented as different product subsystems in the $\mathcal{H}^{\otimes N}$ space) will be correlated.

Assuming that LO fluctuations have finite correlations in time we can expect that QAVAR can be efficiently

calculated using a tensor network in the form of a chain of length N with $(d-1)$-dimensional physical indices on each site. Apart from a clear numerical efficiency advantages, the use of tensor networks approach also allows us to constrain the class of input states to be product (bond dimension $D_\psi = 1$) which corresponds to the typical situation in which atomic samples in different time steps are independent of each other, as they prepared anew at the beginning of each interrogation step—note that we will still consider entanglement between physical atoms with which the LO interacts at a given interrogation time step.

As mentioned in the main text the LO fluctuations can be characterized by the autocorrelation function $R(t)$

$$R(t) = \alpha \mathrm{e}^{-\gamma t} + \beta\delta(t), \tag{11}$$

where we can interpret parameters α, β as strength of respectively OU process and white noise and $1/\gamma$ as OU correlation range. We choose $\alpha = 1\,\mathrm{rad}^2\mathrm{s})^{-2}$, $\beta = 0.1\,\mathrm{rad}^2\mathrm{s})^{-1}$, $\gamma = 2\,\mathrm{s}^{-1}$ for which noise correlations on the time scale that will correspond to the optimal interrogation time step T (which in this case will happen to be around 1.3 s) will be weak enough so that they will appreciable affect only the nearest-neighbour "time-step subsystems"—studying noise with further correlations is possible but would require more computational resources because of the larger bond dimensions. For such a noise the AVAR of the free running LO reads:

$$\sigma^2_{\mathrm{LO}}(\tau) = \frac{1}{\tau\omega_0^2}\left[\frac{2\alpha}{\gamma} + \frac{\alpha}{\gamma^2\tau}\left(4\mathrm{e}^{-\gamma\tau} - \mathrm{e}^{-2\gamma\tau} - 3\right) + \beta\right], \tag{12}$$

and in the most interesting regime of large averaging times takes the form $\sigma^2_{\mathrm{LO}}(\tau) \simeq (2\alpha\gamma^{-1} + \beta)/(\tau\omega_0^2)$. We expect that in this limit QAVAR also takes the form $\sigma^2_{\mathrm{Q}}(\tau) \simeq c/(\tau\omega_0^2)$ with some constant c which we will refer to as asymptotic coefficient.

Going back to Fig. 2(a) of the main text we see that the QAVAR curve flatten for $\tau \gtrsim 50\,\mathrm{s}$, which when taking into account the optimal interrogation times which in this case approaches $\sim 1.3\,\mathrm{s}$, implies that for calculations of the QAVAR we would need to consider ~ 80 interrogation steps and hence if full Hilbert space description was used for this purpose would require 2^{80} dimensional space—clearly an impossible task. Similarly as in the previous example, we may directly access the asymptotic behaviour ($\tau \to \infty$) of the QAVAR function with the help of the iMPO approach. Note that, the definition of the AVAR (23) leads to an intrinsically not translationally invariant MPO for $\underline{\rho}'$ in the expression for QAVAR. Because of this when implementing the iMPO approach we approximate AVAR by an asymptotically equivalent expression:

$$\sigma^2(\tau) \simeq \frac{1}{\tau^2\omega_0^2}\left\langle\left(\int_0^\tau \mathrm{d}t\,\omega(t)\right)^2\right\rangle, \tag{13}$$

which coincide with previous definition for τ much larger then the noise correlation length (which is exactly the regime in which iMPO approach operates).

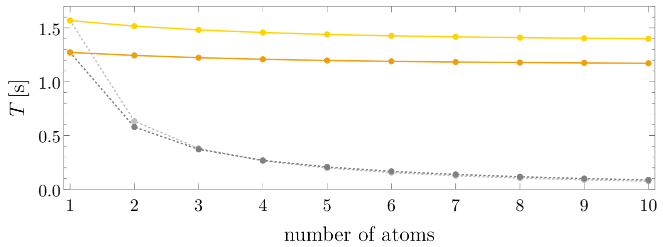

Supplementary Figure 4. Optimal interrogation times. Optimal interrogation times as a function of the number of atoms in the atomic clock with LO noise which is strictly local (yellow dots connected by solid line/light grey dots connected by dotted line) or also includes the nearest neighbours correlations (orange dots connected by solid line/grey dots connected by dotted line) for the optimal/NOON state.

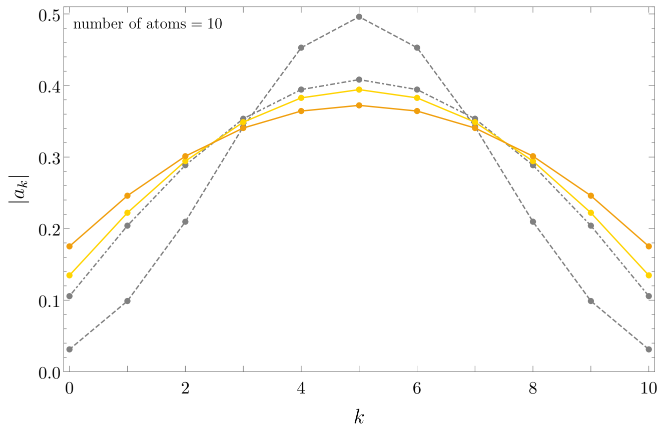

Supplementary Figure 5. Probability amplitudes for the optimal states. Absolute values of the probability amplitudes for the optimal states in the atomic clock (based on 10 atoms) with LO noise which is strictly local (yellow dots connected by solid line) or also includes the nearest neighbours correlations (orange dots connected by solid line), plotted against coherent spin state (CSS, grey dashed line) and the sine state[15]—optimal in phase estimation in case of a completely unknown phase (grey dash-dotted line).

As an add-on to the Fig. 2(b) where the optimal asymptotic QAVAR coefficients are plotted in Supplementary Figure 4 we plot the corresponding optimal interrogation times, which again indicates that presence of noise correlations makes a difference for the values of optimal interrogation times.

Finally, our framework allow us also to easily study the optimal input states. In Supplementary Figure 5 we plot the absolute values of the probability amplitudes a_k for the optimal states (for LO noise which is strictly local or also includes the nearest neighbours correlations) alongside a_k for coherent spin state (CSS) and the sine

state[15] (which is the optimal input state for Bayesian estimation of phase with a flat prior distribution). We see that our optimal states are nonclassical, which we can quantify by calculating their spin-squeezing parameter $\xi^2 = 2\Delta^2 J_y/\langle J_x\rangle$[14] which is 1 for CSS, 0.522 for sine state, 0.456 and 0.378 for the optimal states for LO noise which is respectively strictly local or also includes the nearest neighbours correlations.

SUPPLEMENTARY NOTE 4. REVIEW OF TENSOR NETWORKS

Here we give a short review of the tensor-network formalism (for a more comprehensive recent review see[16]). To succinctly describe tensor networks we represent tensors diagrammatically: suppose that T is a tensor with N indices, each ranging from 0 to $d-1$, elements of this tensor we denote as $T_{j_1 j_2 \dots j_N}$. We depict such a tensor as a circle with N legs, each labelled with an index:

$T_{j_1 j_2 \dots j_N} =$ 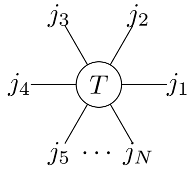

.

Tensors in this paper are nothing more than lists of numbers; we don't assume any particular transformation properties for our tensors. Some simple special cases include kets $|v\rangle$, bras $\langle v|$ ($N = 1$) and operators $\mathcal{M}$ ($N = 2$):

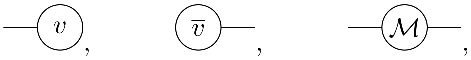

where the overline denotes complex conjugation.

Tensor contraction, whereby the components of two tensors T and W are multiplied and summed over repeated indices, is depicted by connecting the legs, e.g.,

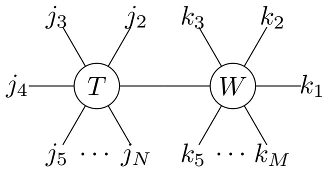

.

Here this tensor network depicts the contraction

$$\sum_{l=0}^{d-1} T_{l j_2 \dots j_N} W_{k_1 \dots k_3 l k_5 \dots k_M}, \tag{14}$$

which is a tensor with $N+M-2$ legs. By combining tensors with three or more legs via tensor contraction we can build networks of arbitrary complexity. (Tensor networks involving tensors with one or two legs are necessarily a combination of lines and cycles.)

Among various tensor network classes, the most important for this paper are the matrix product state (MPS) and matrix product operator (MPO) tensor networks. MPS/MPO are a natural compact representation for states/operators with finite correlations so we expect that they form natural language to study quantum metrology problems with locally correlated noise. An MPS with open boundary conditions (OBC) is a state of the form

$|\psi\rangle =$ 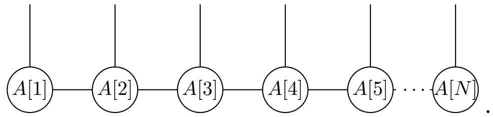

.

Matrix product states with OBCs are now known to provide an excellent model for the ground states of one-dimensional quantum spin chains with (constant) spectral gap[17].

We can accommodate periodic boundary conditions (PBCs) by joining the last tensor to the first via an additional "horizontal leg":

$|\psi\rangle =$ 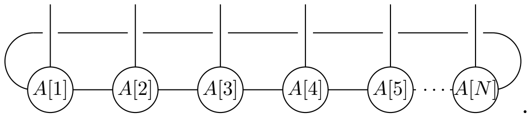

.

Such MPS with PBCs offer some numerical advantages for quantum spin systems on rings[18].

A matrix product operator (MPO) is a tensor network which is, in the PBCs case, a linear operator from $\left(\mathbb{C}^d\right)^{\otimes N}$ to itself parametrised according to

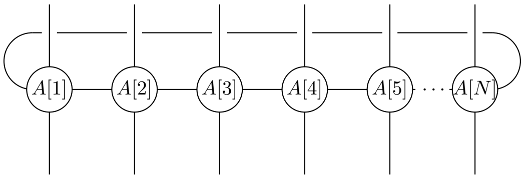

,

where the last horizontal leg is contracted with the first. Here the tensor $A[n]$ has four legs:

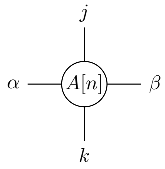

.

The horizontal-leg indices (also called the virtual indices) α and β range from $1, 2, \dots, D$, where the parameter D is called the bond dimension and the vertical leg indices (also called the physical indices) j and k range from $0, 1, \dots, d-1$, where d is the physical dimension. Thus an MPO μ with PBCs is the following operator

$$\mu = \sum_{\substack{j_1,\dots,j_N,\\ k_1,\dots,k_N=0}}^{d-1} \mathrm{Tr}\left(A[1]^{j_1}_{k_1} A[2]^{j_2}_{k_2} \dots A[N]^{j_N}_{k_N}\right) \times |j_1, j_2, \dots, j_N\rangle\langle k_1, k_2, \dots, k_N|. \tag{15}$$

Exploiting channel/state duality we can bend the vertical legs upward ($|j\rangle\langle k| \to |j,k\rangle$) to write an MPO μ as an MPS $|\mu\rangle$ of $2N$ particles:

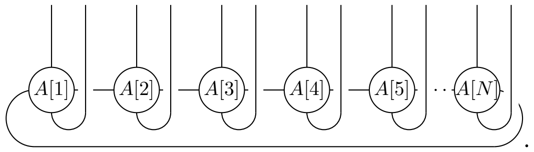

Defining a new vertical line $\mathbf{|} = ||$ to range over a doubled index (j,k), with $j,k = 0,1,\ldots,d-1$, we arrive at the equivalent tensor network for $|\mu\rangle$:

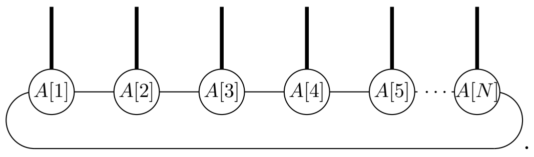

The multiplication of two MPOs μ and ν, with bond dimensions D_1 and D_2, respectively, is given by

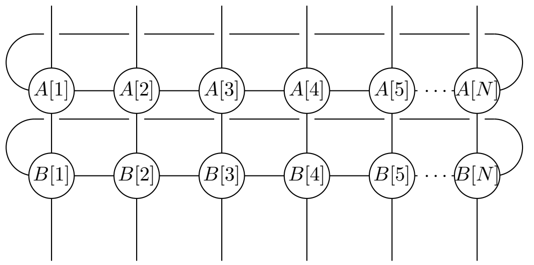

Contracting the two tensors A and B vertically, and combining the two horizontal lines into a new horizontal line ranging from $1,2,\ldots,D_1D_2$:

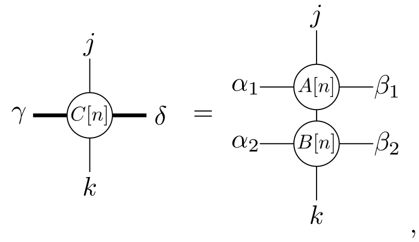

results in a new MPO $\mu\nu$ with bond dimension D_1D_2:

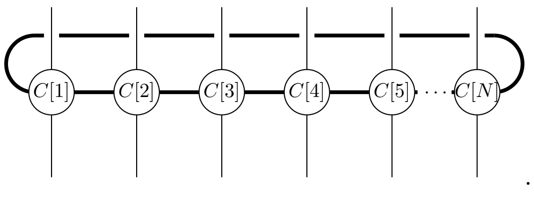

The bond dimension D is a refinement parameter limiting the correlations occurring in the MPO/MPS representation of an operator/state: when the correlations have a finite range there is a finite D capable of representing operator/state accurately. As the cost of tensor network computations is polynomial in D, the MPO/MPS ansatz is a basis for powerful numerical methods. The bond dimension of an MPS is directly connected to the entanglement between a bipartition of the chain: when one computes the entanglement entropy of a contiguous collection $[j,k] = \{j, j+1, \ldots, k\}$ of spins one may derive the bound

$$S(\rho_{[j,k]}) \le 2\log_2(D), \tag{16}$$

on the entanglement between the region $[j,k]$ and the rest of the chain (see, e.g.,[19–21] for an elaboration of this result amongst many others). Accordingly, if a quantum state has a large bipartite entanglement then a larger D is required to represent it as an MPS, and hence the harder it is to approximate it numerically.

A central tool for tensor network manipulations is the singular value decomposition (SVD), according to which, for all operators T there exist unitaries U and V and a diagonal matrix S with non-negative real numbers on the diagonal called singular values, such that

$$T = USV^{\dagger}. \tag{17}$$

This may be diagrammatically represented as follows:

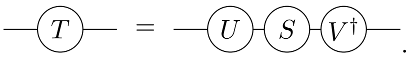

So far we have discussed tensor networks for finite collections of particles. A crucial advantage of the tensor-network formalism is that we can easily extend the MPO (as well as MPS) ansatz to apply to infinite-sized systems; we simply allow the network to extend to infinity from either side:

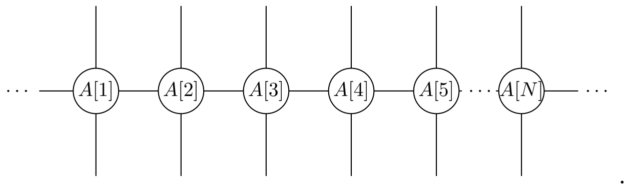

In order to work with such networks it is expedient to assume translation invariance (TI), which is imposed by assuming the tensor does not vary from site to site, so for each n: $A[n] = A$. With this simple assumption it becomes possible to contract and evaluate infinite MPO (iMPO).

A key primitive operation for manipulations involving iMPOs is the trace. Diagrammatically the trace $\mathrm{Tr}(\mu)$ of an iMPO may be obtained by connecting the vertical legs:

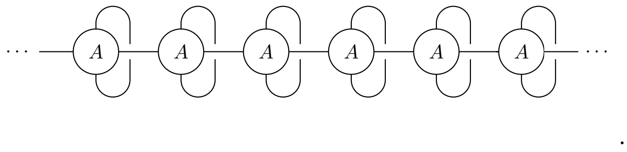

Define

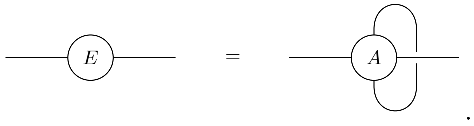

In this way we obtain for the trace $\mathrm{Tr}(\mu)$ a tensor network involving the infinite product of so-called transfer matrices E:

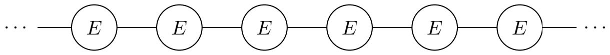

or, in equations, $\mathrm{Tr}(\mu) = \lim_{n\to\infty} \mathrm{Tr}(E^n)$. To calculate this expression we note that when E is diagonalizable (does not have to be Hermitian) we can decompose it: $E = \sum_j \lambda_j |r_j)(l_j|$, where $|r_j)$, $(l_j|$ are respectively right and left eigenvectors of E which can be normalized that $(l_i|r_j) = \delta_{ij}$. This decomposition in diagrammes looks like

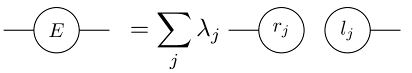

and give us dominant contribution of E^n, determined by the (here assumed unique) leading eigenvalue λ_1:

$$E^n \sim \lambda_1^n |r_1)(l_1| \tag{18}$$

and $\mathrm{Tr}(E^n) \sim \lambda_1^n$. Thus, the limit in an expression such as $\lim_{n\to\infty} \mathrm{Tr}(E^n)/\lambda_1^n$ exists and is equal to 1.

REFERENCES

[1] Heinz-Peter Breuer and Francesco Petruccione, *The Theory of Open Quantum Systems* (Oxford University Press, 2002).

[2] H. F. Trotter, "On the product of semi-groups of operators", Proc. Amer. Math. Soc. **10**, 545–551 (1959).

[3] Masuo Suzuki, "Pair-product model of heisenberg ferromagnets", Journal of the Physical Society of Japan **21**, 2274–2290 (1966).

[4] Masuo Suzuki, "Relationship between d-dimensional quantal spin systems and (d+1)-dimensional ising systemsequivalence, critical exponents and systematic approximants of the partition function and spin correlations", Progress of Theoretical Physics **56**, 1454–1469 (1976).

[5] U. Dorner, R. Demkowicz-Dobrzanski, B. J. Smith, J. S. Lundeen, W. Wasilewski, K. Banaszek, and I. A. Walmsley, "Optimal quantum phase estimation", Phys. Rev. Lett. **102**, 040403 (2009).

[6] B. M. Escher, R. L. de Matos Filho, and L. Davidovich, "General framework for estimating the ultimate precision limit in noisy quantum-enhanced metrology", Nat. Phys. **7**, 406 (2011).

[7] Rafal Demkowicz-Dobrzanski, Jan Kolodynski, and Madalin Guta, "The elusive heisenberg limit in quantum-enhanced metrology", Nat. Commun. **3**, 1063 (2012).

[8] Marcin Jarzyna and Rafał Demkowicz-Dobrzański, "Matrix product states for quantum metrology", Phys. Rev. Lett. **110**, 240405 (2013).

[9] J. Ignacio Cirac, Didier Poilblanc, Norbert Schuch, and Frank Verstraete, "Entanglement spectrum and boundary theories with projected entangled-pair states", Phys. Rev. B **83**, 245134 (2011).

[10] Rafal Demkowicz-Dobrzański and Lorenzo Maccone, "Using entanglement against noise in quantum metrology", Phys. Rev. Lett. **113**, 250801 (2014).

[11] Rafał Demkowicz-Dobrzański, Jan Czajkowski, and Pavel Sekatski, "Adaptive quantum metrology under general markovian noise", Phys. Rev. X **7**, 041009 (2017).

[12] Sisi Zhou, Mengzhen Zhang, John Preskill, and Liang Jiang, "Achieving the heisenberg limit in quantum metrology using quantum error correction", Nat. Commun. **9**, 78 (2018).

[13] Jan Czajkowski, Krzysztof Pawłowski, and Rafał Demkowicz-Dobrzański, "Many-body effects in quantum metrology", New J. Phys. **21**, 053031 (2019).

[14] Jian Ma, Xiaoguang Wang, C.P. Sun, and Franco Nori, "Quantum spin squeezing", Physics Reports **509**, 89 – 165 (2011).

[15] D. W. Berry and H. M. Wiseman, "Optimal states and almost optimal adaptive measurements for quantum interferometry", Phys. Rev. Lett. **85**, 5098–5101 (2000).

[16] Jacob C Bridgeman and Christopher T Chubb, "Hand-waving and interpretive dance: an introductory course on tensor networks", Journal of Physics A: Mathematical and Theoretical **50**, 223001 (2017).

[17] M B Hastings, "An area law for one-dimensional quantum systems", Journal of Statistical Mechanics: Theory and Experiment **2007**, P08024–P08024 (2007).

[18] F. Verstraete, D. Porras, and J. I. Cirac, "Density matrix renormalization group and periodic boundary conditions: A quantum information perspective", Phys. Rev. Lett. **93**, 227205 (2004).

[19] M. Fannes, B. Nachtergaele, and R. F. Werner, "Finitely correlated states on quantum spin chains", Comm. Math. Phys. **144**, 443–490 (1992).

[20] Guifré Vidal, "Efficient classical simulation of slightly entangled quantum computations", Phys. Rev. Lett. **91**, 147902 (2003).

[21] Guifré Vidal, "Efficient simulation of one-dimensional quantum many-body systems", Phys. Rev. Lett. **93**, 040502 (2004).
